# Mobile Phone Dependence, Social Support and Impulsivity in Chinese University Students

**DOI:** 10.3390/ijerph15030504

**Published:** 2018-03-13

**Authors:** Songli Mei, Jingxin Chai, Shi-Bin Wang, Chee H. Ng, Gabor S. Ungvari, Yu-Tao Xiang

**Affiliations:** 1 School of Public Health, Jilin University, Changchun 130021, China; meisongli@sina.com; 2 Hepin Street, Dongcheng District, Beijing Center for Disease Prevention and Control, Beijing 100013, China; 3 Guangdong Mental Health Center, Guangdong General Hospital and Guangdong Academy of Medical Sciences, Guangzhou 510120, China; spiriorwang@126.com; 4 Department of Psychiatry, University of Melbourne, Melbourne, VIC 3050, Australia; cng@unimelb.edu.au; 5 Division of Psychiatry, Notre Dame university Australia, Fremantle, WA 6160, Australia; sungvari@e.cuhk.edu.hk; 6 Graylands Hospital, Claremont, WA 6010, Australia; 7 Unit of Psychiatry, Faculty of Health Sciences, University of Macau, Avenida da Universidade, Taipa, Macau, China

**Keywords:** mobile phone dependence, mobile phone use, impulsivity, China

## Abstract

This study examined the frequency of mobile phone dependence in Chinese university students and explored its association with social support and impulsivity. Altogether, 909 university students were consecutively recruited from a large university in China. Mobile phone use, mobile phone dependence, impulsivity, and social support were measured with standardized instruments. The frequency of possible mobile phone use and mobile phone dependence was 78.3% and 7.4%, respectively. Multinomial logistic regression analyses revealed that compared with no mobile phone dependence, possible mobile phone dependence was significantly associated with being male (*p* = 0.04, OR = 0.7, 95% CI: 0.4–0.98), excessive mobile phone use (*p* < 0.001, OR = 1.2, 95% CI: 1.09–1.2), and impulsivity (*p* < 0.001, OR = 1.05, 95% CI: 1.03–1.06), while mobile phone dependence was associated with length of weekly phone use (*p* = 0.01, OR = 2.5, 95% CI: 1.2–5.0), excessive mobile phone use (*p* < 0.001, OR = 1.3, 95% CI: 1.2–1.4), and impulsivity (*p* < 0.001, OR = 1.08, 95% CI: 1.05–1.1). The frequency of possible mobile phone dependence and mobile phone dependence was high in this sample of Chinese university students. A significant positive association with impulsivity was found, but not with social support.

## 1. Introduction

Mobile phone dependence, defined as inapt use of a mobile phone, is broadly viewed as a subset of behavioral or technological addiction [1] which could lead to significant social and emotional impairment [2]. Excessive mobile phone use is common among young people and is negatively associated with academic performance [3], interpersonal relationships, self-esteem, self-regulation, and life satisfaction [4].

High level of impulsivity appears to be closely associated with excessive mobile phone use and dependence [5,6]. For example, Smetaniuk [7] reported that mobile phone dependence could be considered as an impulse control disorder. Those with a high level of impulsivity often prefer mobile phone use as a choice of gratification without fully thinking through consequences of the action [6]. In addition, social extraversion has been shown to have a positive association with mobile phone addiction, while self-esteem has a negative association [8]. Furthermore, social support is negatively associated with mobile phone use [9], but frequent social networking could increase the risk of mobile phone addiction [10].

China has become the largest mobile phone market worldwide and mobile phone use has increased dramatically in Chinese university students in recent years [11]. Better understanding of the frequency of mobile phone dependence and its associated factors is important to develop effective strategies to reduce its harmful impact. To date, few studies have examined the association of mobile phone dependence with impulsivity and social support. In this study, we aimed to examine the frequency of mobile phone dependence in Chinese university students and explore its association with demographic variables, impulsivity, and social support.

## 2. Materials and Methods

### 2.1. Study Design and Participants

This cross-sectional study was conducted in a large university in Jilin province, China between 1 September and 30 November 2013. Students who fulfilled the following inclusion criteria were recruited: (1) being registered as undergraduate students; (2) having a mobile phone; (3) having the ability to complete the assessment; (4) having the willingness to participate in the study and provide informed consent. The study protocol was approved by the Human Ethical Committee of the Jilin University School of Public Health (BSERE-APP002-FHS).

Three of the six campuses in the University were randomly selected and students who were enrolled in the selected campuses were consecutively screened for eligibility during the study period following a detailed explanation about the study protocol. The questionnaires were distributed to all eligible students in person by the research team members after they provided informed consent. Completed questionnaires were collected on a voluntary basis on the same day. The survey was completed anonymously and confidentially.

### 2.2. Assessment Instruments

Basic demographic characteristics were recorded on a standard study form. At the time of the study, no validated standardized questionnaires on mobile phone use were available in China. Therefore a self-reported questionnaire entitled “Mobile Phone Use Questionnaire (MPUQ)” was developed based on the recommendation of the China Internet Network Information Center [12]. The MPUQ consists of two parts. In the first part, respondents were screened for smart phone use by asking: “Do you own and use a smart phone?” If the answer was “yes”, then the respondent was required to complete the second part. In the second part, the frequency of mobile phone use was assessed in two domains: (1) “use for networking purposes”, including voice calling, sending short messages, using communication programs, watching internet news and blogs; (2) “use for entertainment purposes”, including listening to music, watching videos, playing online games, and reading internet novels. Each domain includes four items with each item scoring from 1 (never) to 4 (frequently). The MPUQ total score ranges from 8 to 32, with a higher score indicating more frequent mobile phone use. The details of MPUQ items are presented in Supplementary Materials Table S1. The internal consistency of the MPUQ was fair: Cronbach’s α = 0.66 for the whole scale, and 0.68 and 0.58 for the two parts, respectively.

The Chinese version of the Barratt Impulsiveness Scale (BIS-11) was used to measure impulsivity trait [13]. The BIS-11 is a 30-item self-reported questionnaire with three subscales: attention impulsivity, motor impulsivity, and non-planning impulsivity. Each item is scored from 1 (never) to 5 (always). The Chinese version of the BIS-11 has been validated with good reliability and validity in China [14]. The internal consistency of the BIS-11 was satisfactory (Cronbach’s α = 0.87) in this study.

The factor structure of Chinese version of the BIS-11 was tested using the confirmatory factor analysis (CFA) with the AMOS 18.0 program. The model was acceptable if the value of *χ*^2^/*df* was less than 8, the goodness of fit index (GFI) was greater than 0.90, the comparative fit index (CFI) was greater than 0.90, or the root mean square of approximation (RMSEA) was less than 0.08 [15]. The factor structure of Chinese version of the BIS-11 was found to be acceptable (*χ*^2^/*df* = 7.42, *GFI* = 0.95, *CFI* = 0.89, *RMSEA* = 0.08). 

The multidimensional scale of perceived social support (MSPSS) is a self-reported questionnaire measuring perceived social support. There are three subscales with a total of 12 items: family members’ support, friends’ support, and others’ support. The items is scored from 1 (strongly disagree) to 7 (strongly agree). The Chinese version of the MSPSS has been validated in China [16]. The internal consistency of the MSPSS in this study was satisfactory (Cronbach’s α = 0.83). The factor structure of Chinese version of the MSPSS was found to be acceptable (*χ*^2^/*df* = 7.81, *GFI* = 0.93, *CFI* = 0.94, *RMSEA* = 0.08).

Mobile phone addiction was assessed using the mobile phone addiction scale for college students (MPAS) [17]. The MPAS comprises 16 items in four areas: withdrawal symptoms, salience, social comfort, and mood changes. Each item is rated on a five-point (1–5) scale with higher scores indicating higher level of mobile phone addiction. A total score of 16–31 indicates “no mobile phone addiction”, while the score of 32–56 indicates “possible mobile phone addiction”, and 57 or above indicates “mobile phone addiction” [9]. This definition of mobile phone addiction was also used in a previous study [16]. The factor structure of the MPAS was found to be acceptable (*χ*^2^/*df* = 2.92, *NFI* = 0.94, *CFI* = 0.96, *RMSEA* = 0.07).

### 2.3. Statistical Analysis

The database was established using EpiData 3.2 (Epidata Assoc., Odense, Denmark). The data were analyzed with SPSS 18.0 (SPSS, Chicago, IL, USA) for Windows. Comparisons between no dependence, possible dependence, and mobile phone dependence individuals with respect to demographic and mental variables were conducted with chi-square and analysis of variance (ANOVA), as appropriate. Multinomial logistic regression analysis was used to determine the independent correlates significantly associated with mobile phone dependence. Mobile phone dependence was the dependent variable, while the demographic and clinical characteristics that significantly differed in the univariate analyses were entered as independent variables. Statistical significance was set at 0.05 (two-tailed).

## 3. Results

Out of 946 students who were approached, 909 students consented to the study and completed the questionnaire, giving a response rate of 96.1%. The frequency of having no mobile phone dependence, possible mobile phone dependence, and mobile phone dependence was 14.3% (*n* = 130), 78.3% (*n* = 712), and 7.4% (*n* = 67), respectively.

Table 1 presents the socio-demographic and clinical characteristics of the whole sample and separately by groups of mobile phone dependence. Univariate analyses revealed that being a single child, weekly mobile phone use time, and the total scores of the MPUQ and BIS were significantly different between the three groups.

Multinomial logistic regression analyses revealed that compared with no mobile phone dependence, possible mobile phone dependence was significantly associated with male gender (*p* = 0.04, OR = 0.7, 95% CI: 0.4–0.98) and MPUQ (*p* < 0.001, OR = 1.2, 95% CI: 1.09–1.2) and BIS total scores (*p* < 0.001, OR = 1.05, 95% CI: 1.03–1.06), while mobile phone dependence was associated with length of weekly phone use (*p* = 0.01, OR = 2.5, 95% CI: 1.2–5.0) and MPUQ (*p* < 0.001, OR = 1.3, 95% CI: 1.2–1.4) and BIS total scores (*p* < 0.001, OR = 1.08, 95% CI: 1.05–1.1) (see Table 2). Supplementary Materials Table S1 shows the details regarding the purpose of mobile phone use. 

## 4. Discussion

In this study, 78.3% and 7.4% of university students reported possible mobile phone dependence and mobile phone dependence, respectively, which is inconsistent with previous findings using the MPAS in Chinese young adults (possible mobile phone dependence: 56.4%; mobile phone dependence: 36.4%) [9] and British and American college students (mobile phone dependence 10–25%) [7,18]. The discrepancy in the results across studies could be due to different socioeconomic and cultural factors, such as age group [7], type of university [11], and religion [19]. The major purpose of mobile phone use in this sample was voice calling and sending short messages, followed by using communication programs, watching internet news, reading blogs, listening music, watching video, playing online games, and reading internet novels.

Compared to male students, female students reported more frequent possible mobile phone dependence, but there was no significant gender difference in mobile phone dependence, which is partly supported by earlier findings in other settings [20,21]. This could be related to gender preferences in the use of mobile phones for maintaining interpersonal relationship [19]. Similar to previous studies [19,22] although different measures were used, we found that excessive use of mobile phones was positively associated with mobile phone dependence.

We found that impulsivity was positively associated with both possible mobile phone dependence and mobile phone dependence, which is in line with previous studies. For example, Smetaniuk [7] found a positive relationship between problematic mobile phone use and impulse control. Roberts et al. [23] found that attention impulsivity is predictive of mobile phone addiction. Further qualitative and empirical studies are warranted to explore the association between mobile phone behavior and impulsivity.

A mobile phone is a communication tool that can enhance social support. Therefore, greater family support could increase the likelihood of excessive mobile phone use [24], which may increase the risk of mobile phone dependence. For example, Crosswhite et al. [25] found that young adults prefer texting to engaging in general conversation with their parents. On the other hand, mobile phone dependence could negatively affect social relationships which in turn may have a negative impact on academic performance [26]. However, our study found no significant association between social support and mobile phone dependence in this study. 

There are several limitations in this study. First, this was a cross-sectional study, therefore the causality between mobile phone dependence and other variables could be not examined. Second, the study was conducted only in one university so the findings could not be generalized to all Chinese university students. Multicenter studies involving different regions across China are needed. Third, other important information related to mobile phone dependence—such as depressive and anxiety symptoms—were not recorded. Fourth, second-digital divide and third-digital divide which may affect individual behavior including impulsiveness, was not examined in this study. Fifth, the mediating effects of various purposes of mobile phone use on impulsiveness could not be examined due to the current study design. Finally, the Cronbach’s α of the MPUQ was relatively low.

## 5. Conclusions

In conclusion, the frequency of possible mobile phone dependence and mobile phone dependence in this sample of Chinese university students was relatively high. A significant positive association between mobile phone dependence and impulsivity was found. Further longitudinal research is needed to explore the relationship between mobile phone dependence and impulsivity, and how the different purposes for mobile phone use could mediate their interaction.

## Figures and Tables

**Table 1:** Comparison of socio-demographic and clinical characteristics between different groups of phone dependence.

	Whole Sample (*n* = 909)		No Phone Dependence (*n* = 130)		Possible Phone Dependence (*n* = 712)		Phone Dependence (*n* = 67)		Statistics ^a^		
	*n*	%	*n*	%	*n*	%	*n*	%	*χ*^2^	*df*	*p*
Male	404	44.4	70	53.8	302	42.5	31	46.3	5.9	2	0.053
Grade											
UG1-UG4	644	70.8	88	67.7	508	71.3	48	71.6	3.7	4	0.4
UG5	132	14.5	24	18.5	96	13.5	12	17.9			
PG	133	14.6	18	13.8	108	15.2	7	10.4			
Urban	426	46.9	63	48.5	330	46.3	33	49.3	0.4	2	0.8
Single child	485	53.4	66	50.8	373	52.4	46	68.7	6.9	2	**0.03**
Length of phone use >3 years	647	71.2	95	73.1	503	70.6	49	73.1	0.45	2	0.8
Length of weekly phone use >24 h	512	56.4	55	42.3	407	57.2	50	74.6	19.8	2	**<0.001**
	**Mean**	**SD**	**Mean**	**SD**	**Mean**	**SD**	**Mean**	**SD**	***f***	***df***	***p***
MPUQ total	26.0	5.1	23.0	5.2	26.2	4.8	29.7	5.6	44.7	2	**<0.001**
BIS total	73.8	12.9	67.7	13.2	74.4	12.7	78.8	11.5	21.0	2	**<0.001**
PSS total	64.4	11.1	64.0	13.1	64.2	10.8	66.8	9.9	1.8	2	0.2

Bolded values: <0.05; ^a^ comparison between no, possible and phone dependence; BIS = Barratt Impulsiveness Scale; PSS = perceived social support; PG = postgraduate; MPUQ = Mobile Phone Use Questionnaire; UG = under graduate.

**Table 2:** Factors independently associated with phone dependence (multinomial logistic regression analysis with no dependence as the reference group).

	Possible Phone Dependence vs. No Dependence			Phone Dependence vs. No Dependence		
	*p*	OR	95% CI	*p*	OR	95% CI
Male	**0.04**	**0.7**	**0.4, 0.98**	0.6	0.8	0.5, 1.6
Single child	0.9	1.0	0.7, 1.5	0.2	1.6	0.8, 3.2
Length of weekly phone use >24 h	0.1	1.4	0.9, 2.1	**0.01**	**2.5**	**1.2, 5.0**
MPUQ total	**<0.001**	**1.2**	**1.09, 1.20**	**<0.001**	**1.3**	**1.2, 1.4**
BIS total	**<0.001**	**1.05**	**1.03, 1.06**	**<0.001**	**1.08**	**1.05, 1.1**

Bolded value: <0.05; BIS = Barratt Impulsiveness Scale; MPUQ = Mobile Phone Use Questionnaire; OR = odds ratio.

## Supplementary Materials

The following are available online at http://www.mdpi.com/1660-4601/15/3/504/s1, Table S1: Factor loadings of mobile phone use questionnaire (MPUQ).
