# Supplementary material for: Mobile Phone Dependence, Social Support and Impulsivity in Chinese University Students
Source: Int J Environ Res Public Health. 2018 Mar 13;15(3):504. doi: 10.3390/ijerph15030504 (PMC5877049; doi:10.3390/ijerph15030504)
Supplement: Supplementary file 1 [file ijerph-15-00504-s001.pdf]

Table S1. Factor loadings of Mobile Phone Use Questionnaire (MPUQ).

	Items	Mean	S.D.	Loading1	Loading2	Loading3
1.	calling and sending short messages	3.30	0.74	0.60	0.01	−0.12
2.	using communication programs	3.29	0.83	0.81	0.08	0.09
3.	watching internet news	3.01	0.90	0.63	0.12	0.12
4.	blogs	3.02	0.96	0.74	0.12	0.17
5.	listening music	3.06	0.89	0.31	−0.13	0.67
6.	watching video	2.37	1.04	0.18	0.24	0.68
7.	playing online games	1.82	0.94	0.08	0.35	0.60
8.	reading internet novels	2.43	1.05	−0.13	0.11	0.58
